# Combination of *Cpb*-*Hsp70* typing methods reveals genetic divergence between *Leishmania infantum* strains causing human tegumentary leishmaniasis in northern Italy and central Spain: a retrospective study

**DOI:** 10.1186/s40249-025-01309-5

**Published:** 2025-05-26

**Authors:** Tommaso Gritti, Carmen Chicharro, Eugenia Carrillo, Jose Carlos Solana, Javier Moreno, Elena Carra, Margherita Ortalli, Sara Morselli, Valeria Gaspari, Margherita Zanazzi, Tiziana Lazzarotto, Gianluca Rugna, Stefania Varani

**Affiliations:** 1 Department of Medical and Surgical Sciences, University of Bologna, 40138 Bologna, Italy; 2 WHO Collaborating Centre for Leishmaniasis, National Centre for Microbiology, Instituto de Salud Carlos III, 28220 Majadahonda, Spain; 3 Centro de Investigación Biomédica en Red de Enfermedades Infecciosas (CIBERINFEC), Instituto de Salud Carlos III, 28029 Madrid, Spain; 4 Istituto Zooprofilattico Sperimentale della Lombardia e dell’Emilia Romagna, 25124 Brescia, Italy; 5 Unit of Microbiology, IRCCS Azienda Ospedaliero-Universitaria di Bologna, 40138 Bologna, Italy; 6 Unit of Dermatology, Head and Neck Department, IRCCS Azienda Ospedaliero-Universitaria di Bologna, 40138 Bologna, Italy

**Keywords:** *Leishmania donovani*, Molecular epidemiology, Cysteine peptidase b, Heat shock protein 70, Tegumentary leishmaniasis

## Abstract

**Background:**

Tegumentary leishmaniasis (TL) caused by *Leishmania infantum* is an overlooked yet re-emerging disease endemic in Mediterranean Europe. Currently, no standardized molecular surveillance of circulating *Leishmania* strains is performed in European endemic areas, despite the potential public health implications of parasite biodiversity. This study aims to characterize parasite population haplogroups causing TL in two active endemic areas in southern Europe, i.e. Bologna (northern Italy) and Fuenlabrada (central Spain).

**Methods:**

In this retrospective study, we typed 87 *L. infantum* samples from TL cases in the areas of Bologna and Fuenlabrada; these areas hosted the main European foci of human TL occurring in the last 15 years. Two *Leishmania* genomic typing targets were used: the heat shock protein 70 *(Hsp70*) and the cysteine peptidase b *(Cpb*). Simpson’s index was used to calculate the discriminatory power of the used typing methods.

**Results:**

Typing results depicted the presence of a heterogeneous parasite population circulating in Bologna with two main haplogroups, i.e. *Hsp70*(A)_*Cpb*(F) (*n* = 7, 30.4%) and *Hsp70*(G)_*Cpb*(E/F) (*n* = 7, 30.4%), differing from the reference *L. infantum* strain JPCM5 haplogroup and partially overlapping with *L. donovani* lineages. Among the samples from Fuenlabrada, *n* = 19 samples were typed by both targets, revealing a homogeneous population expressing *Hsp70*(A) and *Cpb*(E), matching the JPCM5 reference strain haplogroup. Overall, the *Cpb* typing method exhibited higher discrimination power as compared to the *Hsp70* method (Simpson’s index of diversity,* P*-value < 0.05).

**Conclusions:**

Our findings show differences among *L. infantum* populations causing TL in two southern European epidemiological foci of human leishmaniasis and support the recent discovery of *L. infantum/L.donovani* hybrid strains circulating in northern Italy. These results underscore the critical need to identify the circulating *Leishmania* strains in endemic areas and assess their potential public health implications in active foci.

**Graphical Abstract:**

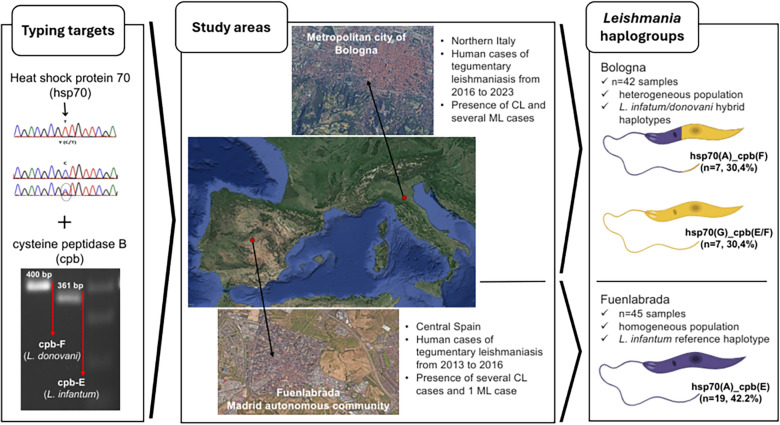

**Supplementary Information:**

The online version contains supplementary material available at 10.1186/s40249-025-01309-5.

## Background

Leishmaniasis is a vector-borne parasitic disease caused by protozoa belonging to the genus *Leishmania* [1]. In the northwestern part of the Mediterranean basin, the disease is caused by the *Leishmania (Leishmania) infantum*, which belongs to the *L. donovani* complex together with *L. donovani* [2]. *L. infantum* can cause the systemic and possibly fatal form of the disease known as visceral leishmaniasis (VL) as well as the localized tegumentary leishmaniasis (TL), including cutaneous (CL) and mucosal leishmaniasis (ML) [3].

In the last fifteen years, two main outbreaks of human leishmaniasis occurred in southern Europe, including the area of Fuenlabrada, close to Madrid in central Spain and the metropolitan city of Bologna in northern Italy. The epidemics occurred in 2009–2012 and 2010–2022, respectively [4, 5]. Characterization of species and strains causing emergence or re-emergence of leishmaniasis in humans is crucial for understanding the population dynamics of circulating parasites and for verifying the potential introduction of allochthonous species or divergent strains [6–8]. Interestingly, *Leishmania* strains isolated from these two epidemic foci exhibited peculiar genetic features different from the main *L. infantum* population defined as Linf1/MON-1 by Franssen et al. [2, 9–12].

Various molecular methods have been proposed for species identification of *Leishmania* strains [7, 13–15]. Among other techniques, the conserved heat shock protein 70 (*Hsp70*) gene is considered a powerful tool to thoroughly identify species belonging to *Leishmania* (*Leishmania)* and *Leishmania (Viannia)*; in particular, a specific sequence of the *Hsp70* gene facilitates population studies, exhibiting single nucleotide polymorphisms (SNP) but no insertion-deletion (indel) polymorphisms [16]. In addition, the cysteine peptidase b (*Cpb*) is considered an accurate target to discriminate the two phylogenetically close species *L. donovani* and *L. infantum*, which are discernable by the presence or absence of a 39 base pair (bp) indel, respectively [7, 15]. These targets were previously employed to genotype *Leishmania* parasites isolated from human cases in northern Italy; *Hsp70* analysis revealed unexpected genetic variation among parasites responsible for TL, including SNP profiles not previously associated with *L. infantum* [11]. In addition, *Cpb* was utilized to characterize *Leishmania* strains from autochthonous VL cases, revealing an amplicon size (400 bp, *type F*) typically linked to *L. donovani*, rather than the size traditionally associated with *L. infantum* (361 bp, *type E*). [17].

Given the importance of parasite biodiversity and its potential public health implications in endemic areas, this study aims to characterize the parasites causing TL in two active endemic areas in southern Europe, i.e., northern Italy and central Spain by studying *Hsp70*-*Cpb* haplogroups.

## Methods

### Study design and study areas

This retrospective study aimed to genotype *Leishmania* parasites in samples from TL-affected patients diagnosed in two different southern European areas. The metropolitan city of Bologna is located within the Emilia-Romagna region in northern Italy and covers an area of 3,703 km^2^, with a population of approximately 1,020,000 inhabitants and a population density of 275.01 inhabitants/km^2^. The second area corresponds to the area served by the Hospital Universitario de Fuenlabrada, defined as Fuenlabrada, which includes approximately 222,500 inhabitants living in the cities of Fuenlabrada, Moraleja de Enmedio and Humanes in the south-west of the Madrid Autonomous Community, in central Spain.

### Case definition and sample collection

The identification of a TL case was based on the World Health Organization (WHO) definition: a patient exhibiting suggestive cutaneous and/or mucosal lesion(s) with confirmation of *Leishmania* parasites or their genome by microscopy and/or molecular methods [3]. CL refers to lesion(s) of skin tissues, while ML refers to mucosal involvement regardless of the co-existence or prior presence of skin lesions. All diagnoses of TL cases from the metropolitan city of Bologna and Fuenlabrada were confirmed by real-time PCR as described by Gaspari et al. [18] and Chicharro et al. [19] for cases from Bologna and Fuenlabrada areas, respectively. The TL specimens included in the study were in total *n* = 87; of these* n* = 80 thin sections from formalin-fixed and paraffin embedded (FFPE) biopsies, *n* = 5 fresh biopsies, and two *Leishmania* strains (MHOM/IT/2023/IZSLER-Bo03 and MHOM/IT/2023/IZSLER-Bo04) obtained from skin/mucosal lesions.

Samples included in the study were collected from confirmed TL cases diagnosed in Bologna from January 1st 2016 to December 30th 2023 and in Fuenlabrada from January 1st 2013 to December 31st 2016. Data about the patients enrolled in the study were anonymized by using an alphanumerical code. The study was conducted in  accordance with the declaration of Helsinki, and the protocol was approved by the Ethics Committee of the Area Vasta Emilia Centro (study number: n° EM414-2023_97/2017/O/Tess/AOUBo) for the Italian cases and by the Fuenlabrada University Hospital (APR12-66 and APR13-30) for the Spanish cases. All participants gave their written, informed consent.

### DNA extraction, amplification and sequence analysis

For samples obtained from Italian TL cases, DNA extraction was performed as follows: the Maxwell® CSC DNA FFPE Kit (Promega, Madison, Wisconsin, United States) was employed in combination with the Maxwell CSC instrument (Promega, Madison, Wisconsin, United States) according to manufacturer instructions for FFPE biopsies, while the DNeasy® Blood&Tissue Kit (Qiagen, Hilden, Germany) was employed to extract DNA from fresh biopsies as well as clinical isolates. For samples obtained from Spanish TL cases, DNA was extracted as described by Chicharro et al. [19] using SpeedTools DNA/Tissue DNA extraction kit (Biotools B&M Labs, S.A., Madrid, Spain) after paraffin removal by treatment with 0.05% Tween 20 in phosphate-buffered saline. PCR reactions were performed using the Thermocycler T-Gradient ThermoBlock (Biometra, Göttingen, Germany) and the HotStarTaq plus kit (Qiagen, Hilden, Germany) according to the manufacturer’s instructions.

Thermal profile, amplification and sequencing conditions of *Hsp70* fragments were performed as described by Gritti et al. [11], with modifications aimed at improving amplification from FFPE samples, which often contain degraded or limited amounts of parasitic DNA. In brief, *Hsp70* was amplified using a three-step nested PCR targeting the N- (593 bp), P- (295 bp), and Ps-fragment (262 bp) of the gene in the first, second, and third amplification rounds, respectively. For each subsequent step, the product from the previous amplification—purified using ExoSAP® (Thermo Fisher, Waltham, MA, USA) following the manufacturer’s instructions—served as template. Thermal cycling conditions for N-fragment amplification were as follows: initial denaturation at 95 °C for 2 min; 35 cycles of 94 °C for 30 s (denaturation), 61 °C for 1 min (annealing), and 72 °C for 1 min (extension); followed by a final extension at 72 °C for 10 min. Amplification of the P- and Ps-fragments followed the same protocol, except for the annealing temperatures, which were set at 62 °C and 63.5 °C, respectively. The primers used for the smallest Ps-fragment amplification were also employed for sequencing all resulting *Hsp70* amplicons. Amplification results were verified by electrophoretic run in a 2% agarose gel stained with GelRed® (Biotium, inc. Fremont, USA) and visualized by UV light. The *Hsp70* consensus sequences were assembled by using the “Sequencing” and “Investigator” packages of GenomeLab™ System Software, version 11.0.24 (Beckman Coulter, Indianapolis, IN, United States). Sequences were identified as *L. donovani* complex spp. by a BLAST search in GenBank at NCBI (https://blast.ncbi.nlm.nih.gov/Blast.cgi, accessed on October 14, 2024) and then aligned with the *L. infantum* reference strain JPCM5 *Hsp70* sequence (XM_001470287) by using Clustal W, as implemented in BioEdit v.7.8.0 [20]. As previously described [11], we designated *Hsp70*(A) as the *Hsp70* sequence corresponding to that of the reference *L. infantum* JPCM5 strain, while *Hsp70(C)*, *Hsp70(E)*, *Hsp70(F)*, and *Hsp70(G)* were identified as variants diverging from the JPCM5 reference sequence.

Amplification of *Cpb* was performed by using primers described by Zackay et al. [13] with thermal conditions as follows: 95 °C for 2 min (activation), followed by 35 cycles at 94 °C for 30 s (denaturation), 65 °C for 30 s (annealing), and 72 °C for 30 s (extension), with a final extension at 72 °C for 10 min. In the case of negative results, a second round of amplification was performed using the same primer set and the previously amplified mix diluted 200 times as sample, and with thermal profile as follows: 95 °C for 2 min (activation), followed by 35 cycles at 94 °C for 30 s (denaturation), 63 °C for 30 s (annealing), and 72 °C for 30 s (extension), with a final extension at 72 °C for 10 min.

To confirm the amplification of the *Cpb* variants, two positive amplification controls were used for each reaction, including genomic DNA of 1) the *L. infantum* reference strain JPCM5 (MCAN/ES/98/LLM-724), generating 361 bp amplicons [defined as *Cpb*(E)], and 2) the *L. donovani* reference strain DD8 (MHOM/IN/80/DD8), whose amplification generates 400 bp long amplicons [defined as *Cpb*(F)] [13]. As positive control, DNA was extracted from cultured promastigotes at a concentration of 1 × 10^4^ parasites/ml. Amplified products were verified by electrophoresis through a 4% agarose/TAE gel stained by soaking, for 30 min, in Ethidium Bromide bath (0.5 µg/ml in water) after the electrophoretic run.

### Statistical analysis

The discriminatory power of the two typing methods was assessed by calculating the Simpson’s index of diversity using a free online tool (http://www.comparingpartitions.info/index.php?link=Tool, accessed on October 14, 2024). *P* values < 0.05 were considered significant.

## Results

### Overall typing results

A total of 87 samples collected from 82 human TL cases were tested; four samples were obtained from two patients who experienced relapses, while six samples were obtained from three patients affected by two lesions each. Of the total, 42 samples [38 TL cases (11 ML and 27 CL)] were from the Bologna area, while 45 samples [45 TL cases (44 CL and 1 ML)] were from the Fuenlabrada area. Of the tested samples, 67 (77.0%) were successfully typed for at least one target, while 42 (48.3%) were typed for both *Hsp70* and *Cpb*. Sample amplification showed an aggregate sensitivity of 71.2% (*n* = 62 amplified samples out of 87) for *Hsp70* and 54.0% (*n* = 47) for *Cpb*, respectively. Accession numbers of the *Hsp70* consensus sequences are shown in Additional file 1 (Table S1), while gel images of the electrophoretic runs are shown in Additional file 2 (Figure S1 and Figure S2).

### Typing results of samples from northern Italy

*Hsp70* was successfully typed in 36 samples out of 42 (85.7%, Table 1); among them, 13 (31%) were identified as sequence variant A [*Hsp70*(A)], 5 (11.9%) as *Hsp70*(C), 17 (40.5%) as *Hsp70*(G) and 1 (2.4%) as *Hsp70*(E). *Cpb* amplification was obtained for 24 samples (57.1%); among them, 3 samples (12.5%) were typed as variant E [*Cpb*(E)], 11 specimens (45.8%) were typed as *Cpb*(F) and 10 samples (41.7%) showed the presence of both amplicon sizes and were therefore defined as *Cpb*(E/F). In all cases with multiple samples from the same patients, the typing results were concordant.

**Table 1:** *Hsp70* and *Cpb* typing in specimens of tegumentary leishmaniasis from the metropolitan city of Bologna (northern Italy, *n* = 42 samples)

	*Cpb*				
	E	E/F	F	N/A	Total
*Hsp70*					
*L. donovani* complex (A)	0	1 (2.4%)	7^a^ (16.7%)	5^d^ (11.9%)	13 (30.9%)
*L. donovani* complex (C)	0	1 (2.4%)	2 (4.8%)	2 (4.8%)	5 (11.9%)
*L. donovani* complex (G)	3^c^ (7.1%)	7 (16.7%)	2 (4.8%)	5^c^ (11.9%)	17 (40.5%)
*L. donovani* complex (E)	0	0	0	1 (2.4%)	1 (2.4%)
N/A	0	1 (2.4%)	0	5^bd^ (11.9%)	6 (14.3%)
Total	3 (7.1%)	10 (23.8%)	11 (26.2%)	18 (42.9%)	42

^a^Two samples with haplogroup *Hsp70*(A)_*Cpb*(F) derived from the same patient

^b^In two samples from the same patient *Cpb* and *Hsp70* could not be amplified

^c^Two samples exhibiting *Hsp70*(G)_*Cpb*(E) and *Hsp70*(G)_*Cpb* (not amplified) respectively, were from the same patient

^d^Two samples exhibiting *Hsp70*(A)_*Cpb*(not amplified), and *Hsp70*(not amplified)_*Cpb*(not amplified),respectively, were from the same patient

N/A; not amplified

Focusing on the samples typed successfully by both targets (*n* = 23) we observed that the most frequent haplogroups were *Hsp70*(A)_*Cpb*(F) (*n* = 7, 30.4%) and *Hsp70*(G)_*Cpb*(E/F) (*n* = 7, 30.4%).

### Typing results of samples from central Spain

*Hsp70* sequence was obtained for 25 samples out of 45 (55.6%, Table 2); all of these were identified as *Hsp70*(A). Furthermore, *Cpb* typing was successfully carried out in 23 samples (51.1%), all presenting the *Cpb*(E) amplicon size. Of all 45 samples, both targets were amplified in 19 samples (42.2%); in this group we identified only one haplogroup that was characterized by *Hsp70*(A)_*Cpb*(E).

**Table 2:** *Hsp70* and *Cpb* typing in specimens of tegumentary leishmaniasis from Fuenlabrada (central Spain, *n* = 45 samples)

	*Cpb*				
	E	E/F	F	N/A	Total
*Hsp70*					
*L. donovani* complex (A)	19 (42.2%)	0	0	6 (13.3%)	25 (55.6%)
*L. donovani* complex (C)	0	0	0	0	0
*L. donovani* complex (G)	0	0	0	0	0
*L. donovani* complex (E)	0	0	0	0	0
N/A	4 (8.9%)	0	0	16 (35.6%)	20 (44.4%)
Total	23 (51.1%)	0	0	22 (48.9%)	45

N/A; not amplified

### Statistical analysis

By considering the samples that were typed successfully by the two methods (*n* = 42), the comparison of the results revealed that the *Cpb* typing method exhibited higher discrimination power as compared to the *Hsp70* method, with a Simpson’s index of diversity of 0.626 (95% *CI*: 0.538–0.714) and 0.512 (95% *CI*: 0.390–0.635), respectively *(P*-value < 0.05).

## Discussion

In this study, we identified genetic differences in clinical strains obtained from TL cases originating from two new epidemic regions: northern Italy and central Spain. By employing *Hsp70* and *Cpb* typing, we found that the parasitic population causing TL in central Spain homogenously exhibited the haplogroup *Hsp70*(A)_*Cpb*(E), which is present in the *L. infantum* reference strain JPCM5, the latter being considered representative of the Linf1/MON-1 population described by Franssen et al. [2, 21]. This is consistent with the established epidemiology of the *L. infantum* zymodeme MON-1 population, which is widely distributed in the canine reservoir throughout the Mediterranean basin and Latin America and is capable of causing both TL and VL in humans [2, 22].

Conversely, the parasitic population responsible for TL in northern Italy exhibited several *Hsp70*-*Cpb* haplogroups, including two predominant ones: *Hsp70*(A)_*Cpb*(F) and *Hsp70*(G)_*Cpb*(E/F). These haplogroups harbor polymorphisms previously associated to *L. donovani*, although this parasitic species has not yet been reported as circulating in Italy [2]. The identification of the *L. donovani*-specific 39 bp undeleted *Cpb* variant F in TL cases from the Bologna area aligns with previous reports of its occurrence in VL cases from the same region [17] and in CL cases from Tunisia [23–25]. In the latter, *Cpb*(F) was detected in *L. infantum* strains belonging to the MON-24 zymodeme, leading the authors to propose a close evolutionary relationship between these strains and the ancestral *L. donovani* complex.

We also detected *Cpb* (E/F) in TL cases from northern Italy; this heterozygous profile can be considered as a *L. infantum/L. donovani* hybrid variant and was previously described in the *L. infantum* reference strain MON-24 (MHOM/DZ/82/LIPA59), isolated from human CL in Algeria [26]. The presence of genetic traces of *L. donovani* in TL strains from northern Italy aligns with a recent whole genome sequencing (WGS) study of VL isolates from the same area, which revealed the circulation of autochthonous *L. infantum/donovani* hybrid strains [12]. Notably, those VL strains were found to carry the *Cpb*(F) allele [17].

The genetic heterogeneity observed among *L. infantum* populations in this study may be associated with different parasite phenotypes, which could correlate with distinct disease presentation, as observed in clinical cases caused by *L. donovani* and *L. major* [27–29].

The retrospective design of the study and the limited number of clinical samples are the main limitations of the present work. Furthermore, the study was designed to focus on *L. infantum* genetic variations that were previously observed in strains circulating in one of the two study sites, specifically northern Italy [11, 17]. Additional genetic markers—such as the Internal Transcribed Spacer-2 or the complete maxicircle coding region, which have previously been used to characterize *L. infantum* strains causing VL in the Madrid area [9, 10]—would be required to accurately characterize the strains causing TL in central Spain. Therefore, by using single-gene typing tools, variations from the classical *L. infantum* genotype that fall outside the studied fragments would not be detected and may cause incorrect interpretation of the results.

Notably, the two target genes of this study, *Cpb* and *Hsp70*, translate for proteins that are related to stress and drug resistance, respectively [30–32]. Considering this fact, we hypothesize that the genomic differences between the parasitic populations causing TL in central Spain and northern Italy may be associated with distinct clinical presentation among TL cases from the two epidemic foci; in Fuenlabrada, mainly CL cases were detected [33], while in Bologna several ML were observed [4, 11, 18, 34]. However, the limited clinical data collected in this study, along with insufficient knowledge of these specific *L. infantum* strains, precludes us from ruling out potential confounding factors and does not allow establishing definitive links between parasitic genotypes and clinical manifestation of the disease. Furthermore, we currently lack sufficient evidence to support a correlation between specific *Hsp70/ Cpb* haplogroups and drug-resistant phenotypes. Thus, additional studies with larger sample groups are needed; these studies could be conducted using selected typing sequences derived from WGS analysis of parasites circulating in the targeted areas.

## Conclusions

The combination of two genomic targets has proven to be a valuable tool for determining strain divergence directly in clinical samples obtained from TL cases in two southern European epidemic foci of human leishmaniasis. Using *Hsp70* and *Cpb* typing, we found that the *Leishmania* population causing TL in central Spain uniformly exhibited the *Hsp70*(A)_*Cpb*(E) haplogroup, characteristic of the *L. infantum* MON-1 zymodeme — the main lineage involved in human and canine infections throughout the Mediterranean region and Latin America. In contrast, the TL-causing strains in northern Italy exhibited greater diversity, with two predominant haplogroups: *Hsp70*(A)_*Cpb*(F) and *Hsp70*(G)_*Cpb*(E/F), both harboring polymorphisms previously linked to *L. donovani*. In conclusion, this study highlights the importance of strengthening genomic surveillance of *Leishmania* strains across the Mediterranean basin through implementation of standardized typing protocols, utilizing single or combined genetic markers.

## Supplementary Information

**Additional file 1. Table S1.** Study dataset and *Hsp70* sequences.

**Additional file 2. Figure S1.** UV light visualization of electrophoretic run of *Cpb*-generated amplicons; **Figure S2.** UV light visualization of electrophoretic run of *Hsp70* N-fragment (593 bp) generated amplicons.

## Data Availability

All data generated during this study are included in this published article [Supplementary Table 1].

## Abbreviations

CL: Cutaneous leishmaniasis
Cpb: Cysteine peptidase b
Hsp70: Heat shock protein 70
Indel: Insertion-deletion
ML: Mucosal leishmaniasis
SNP: Single nucleotide polymorphism
TL: Tegumentary leishmaniasis
VL: Visceral leishmaniasis
WGS: Whole genome sequencing

## References

[1] Akhoundi M, Kuhls K, Cannet A, Votýpka J, Marty P, Delaunay P, et al. A historical overview of the classification, evolution, and dispersion of leishmania parasites and sandflies. PLoS Negl Trop Dis. 2016;10(3): e0004349. 10.1371/journal.pntd.0004349. 26937644 10.1371/journal.pntd.0004349 PMC4777430

[2] Franssen SU, Durrant C, Stark O, Moser B, Downing T, Imamura H, et al. Global genome diversity of the *Leishmania donovani* complex. Elife. 2020;9: e51243. 10.7554/eLife.51243. 32209228 10.7554/eLife.51243 PMC7105377

[3] Gradoni L, López-Vélez R, Mokni M. Manual on Case Management and Surveillance of the Leishmaniases in the WHO European Region. World Health Organization, Danmark. 2017. https://iris.who.int/bitstream/handle/10665/344118/9789289052511-eng.pdf?sequence=1. Accessed 12 April 2025.

[4] Todeschini R, Musti MA, Pandolfi P, Troncatti M, Baldini M, Resi D, et al. Re-emergence of human leishmaniasis in northern Italy, 2004 to 2022: a retrospective analysis. Euro Surveill. 2024;29(4):2300190. 10.2807/1560-7917.ES.2024.29.4.2300190. 38275016 10.2807/1560-7917.ES.2024.29.4.2300190 PMC10986649

[5] Arce A, Estirado A, Ordobas M, Sevilla S, García N, Moratilla L, et al. Re-emergence of leishmaniasis in Spain: community outbreak in Madrid, Spain, 2009 to 2012. Euro Surveill. 2013;18(30):20546. 10.2807/1560-7917.es2013.18.30.20546. 23929177 10.2807/1560-7917.es2013.18.30.20546

[6] Rogers MB, Downing T, Smith BA, Imamura H, Sanders M, Svobodova M, et al. Genomic confirmation of hybridisation and recent inbreeding in a vector-isolated Leishmania population. PLoS Genet. 2014;10(1): e1004092. 10.1371/journal.pgen.1004092. 24453988 10.1371/journal.pgen.1004092 PMC3894156

[7] Van der Auwera G, Dujardin JC. Species typing in dermal leishmaniasis. Clin Microbiol Rev. 2015;28(2):265–94. 10.1128/CMR.00104-14. 25672782 10.1128/CMR.00104-14 PMC4402951

[8] Alvar J, Vélez ID, Bern C, Herrero M, Desjeux P, Cano J, et al. Leishmaniasis worldwide and global estimates of its incidence. PLoS ONE. 2012;7(5): e35671. 10.1371/journal.pone.0035671. 22693548 10.1371/journal.pone.0035671 PMC3365071

[9] Chicharro C, Llanes-Acevedo IP, García E, Nieto J, Moreno J, Cruz I. Molecular typing of *Leishmania infantum* isolates from a leishmaniasis outbreak in Madrid, Spain, 2009 to 2012. Euro Surveill. 2013;18(30):20545. 10.2807/1560-7917.es2013.18.30.20545. 23929179 10.2807/1560-7917.es2013.18.30.20545

[10] Solana JC, Chicharro C, García E, Aguado B, Moreno J, Requena JM. Assembly of a large collection of Maxicircle sequences and their usefulness for Leishmania taxonomy and strain typing. Genes. 2022. 10.3390/genes13061070. 35741832 10.3390/genes13061070 PMC9222942

[11] Gritti T, Carra E, Van der Auwera G, Solana JC, Gaspari V, Trincone S, et al. Molecular typing of *Leishmania* spp. causing tegumentary leishmaniasis in Northeastern Italy, 2014–2020. Pathogens. 2023;13(1):19. 10.3390/pathogens13010019. 38251327 10.3390/pathogens13010019 PMC10820635

[12] Bruno F, Castelli G, Li B, Reale S, Carra E, Vitale F, et al. Genomic and epidemiological evidence for the emergence of a *L. infantum*/*L. donovani* hybrid with unusual epidemiology in northern Italy. Bio. 2024;15(7): e0099524. 10.1128/mbio.00995-24. 10.1128/mbio.00995-24 PMC11253594 38832792

[13] Zackay A, Nasereddin A, Takele Y, Tadesse D, Hailu W, Hurissa Z, et al. Polymorphism in the HASPB repeat region of East African *Leishmania donovani* strains. PLoS Negl Trop Dis. 2013;7(1): e2031. 10.1371/journal.pntd.0002031. 23358849 10.1371/journal.pntd.0002031 PMC3554577

[14] Kuhls K, Mauricio IL, Pratlong F, Presber W, Schönian G. Analysis of ribosomal DNA internal transcribed spacer sequences of the *Leishmania donovani* complex. Microbes Infect. 2005;7(11–12):1224–34. 10.1016/j.micinf.2005.04.009. 16002315 10.1016/j.micinf.2005.04.009

[15] Hide M, Bañuls AL. Polymorphisms of cpb multicopy genes in the *Leishmania (Leishmania) donovani* complex. Trans R Soc Trop Med Hyg. 2008;102(2):105–6. 10.1016/j.trstmh.2007.09.013. 17996911 10.1016/j.trstmh.2007.09.013

[16] Fraga J, Montalvo AM, De Doncker S, Dujardin JC, Van der Auwera G. Phylogeny of *Leishmania *species based on the heat-shock protein 70 gene. Infect Genet Evol. 2010;10(2):238–45. 10.1016/j.meegid.2009.11.007. 19913110 10.1016/j.meegid.2009.11.007

[17] Rugna G, Carra E, Corpus F, Calzolari M, Salvatore D, Bellini R, et al. Distinct Leishmania infantum strains circulate in humans and dogs in the Emilia-Romagna region, Northeastern Italy. Vector Borne Zoonotic Dis. 2017;17(6):409–15. 10.1089/vbz.2016.2052. 28301296 10.1089/vbz.2016.2052

[18] Gaspari V, Gritti T, Ortalli M, Santi A, Galletti G, Rossi A, et al. Tegumentary Leishmaniasis in Northeastern Italy from 2017 to 2020: a neglected public health issue. Int J Environ Res Public Health. 2022;19(23):16047. 10.3390/ijerph192316047. 36498130 10.3390/ijerph192316047 PMC9740434

[19] Chicharro C, Nieto J, Miguelañez S, Garcia E, Ortega S, Peña A, et al. Molecular diagnosis of leishmaniasis in Spain: development and validation of ready-to-use gel-form nested and real-time PCRs to detect. Microbiol Spectr. 2023;11(3): e0335422. 10.1128/spectrum.03354-22. 37014253 10.1128/spectrum.03354-22 PMC10269443

[20] Hall TA. BioEdit: a user-friendly biological sequence alignment editor and analysis program for windows 95/98/NT. Oxford University Press; 1999. p. 95–8.

[21] González-de la Fuente S, Peiró-Pastor R, Rastrojo A, Moreno J, Carrasco-Ramiro F, Requena JM, et al. Resequencing of the *Leishmania infantum* (strain JPCM5) genome and de novo assembly into 36 contigs. Sci Rep. 2017;7(1):18050. 10.1038/s41598-017-18374-y. 29273719 10.1038/s41598-017-18374-y PMC5741766

[22] Kuhls K, Chicharro C, Cañavate C, Cortes S, Campino L, Haralambous C, et al. Differentiation and gene flow among European populations of *Leishmania infantum* MON-1. PLoS Negl Trop Dis. 2008;2(7): e261. 10.1371/journal.pntd.0000261. 18612461 10.1371/journal.pntd.0000261 PMC2438616

[23] Chaouch M, Fathallah-Mili A, Driss M, Lahmadi R, Ayari C, Guizani I, et al. Identification of Tunisian *Leishmania* spp. by PCR amplification of cysteine proteinase B (cpb) genes and phylogenetic analysis. Acta Trop. 2013;125(3):357–65. 10.1016/j.actatropica.2012.11.012. 23228525 10.1016/j.actatropica.2012.11.012

[24] Benikhlef R, Chaouch M, Abid MB, Aoun K, Harrat Z, Bouratbine A, et al. ITS1 and cpb genetic polymorphisms in Algerian and Tunisian* Leishmania infantum* isolates from humans and dogs. Zoonoses Public Health. 2023;70(3):201–12. 10.1111/zph.13016. 36443904 10.1111/zph.13016

[25] Bussotti G, Benkahla A, Jeddi F, Souiaï O, Aoun K, Späth GF, et al. Nuclear and mitochondrial genome sequencing of North-African. Microb Genom. 2020;6(10):mgen000444. 10.1099/mgen.0.000444. 32975503 10.1099/mgen.0.000444 PMC7660250

[26] Hide M, Bañuls AL. Species-specific PCR assay for *L. infantum*/*L. donovani* discrimination. Acta Trop. 2006;100(3):241–5. 10.1016/j.actatropica.2006.10.012. 17141723 10.1016/j.actatropica.2006.10.012

[27] Oryan A, Shirian S, Tabandeh MR, Hatam GR, Randau G, Daneshbod Y. Genetic diversity of Leishmania major strains isolated from different clinical forms of cutaneous leishmaniasis in southern Iran based on minicircle kDNA. Infect Genet Evol. 2013;19:226–31. 10.1016/j.meegid.2013.07.021. 23892374 10.1016/j.meegid.2013.07.021

[28] Mahdi M, Elamin EM, Melville SE, Musa AM, Blackwell JM, Mukhtar MM, et al. Sudanese mucosal leishmaniasis: isolation of a parasite within the *Leishmania donovani* complex that differs genotypically from *L. donovani* causing classical visceral leishmaniasis. Infect Genet Evol. 2005;5(1):29–33. 10.1016/j.meegid.2004.05.008. 15567136 10.1016/j.meegid.2004.05.008

[29] Sreenivas G, Raju BV, Singh R, Selvapandiyan A, Duncan R, Sarkar D, et al. DNA polymorphism assay distinguishes isolates of *Leishmania donovani* that cause kala-azar from those that cause post-kala-azar dermal Leishmaniasis in humans. J Clin Microbiol. 2004;42(4):1739–41. 10.1128/JCM.42.4.1739-1741.2004. 15071036 10.1128/JCM.42.4.1739-1741.2004 PMC387559

[30] Mottram JC, Coombs GH, Alexander J. Cysteine peptidases as virulence factors of *Leishmania.* Curr Opin Microbiol. 2004;7(4):375–81. 10.1016/j.mib.2004.06.010. 15358255 10.1016/j.mib.2004.06.010

[31] Matrangolo FS, Liarte DB, Andrade LC, de Melo MF, Andrade JM, Ferreira RF, et al. Comparative proteomic analysis of antimony-resistant and -susceptible *Leishmania braziliensis* and *Leishmania infantum* chagasi lines. Mol Biochem Parasitol. 2013;190(2):63–75. 10.1016/j.molbiopara.2013.06.006. 23831370 10.1016/j.molbiopara.2013.06.006

[32] Salari S, Bamorovat M, Sharifi I, Almani PGN. Global distribution of treatment resistance gene markers for leishmaniasis. J Clin Lab Anal. 2022;36(8): e24599. 10.1002/jcla.24599. 35808933 10.1002/jcla.24599 PMC9396204

[33] Aguado M, Espinosa P, Romero-Maté A, Tardío JC, Córdoba S, Borbujo J. Outbreak of cutaneous leishmaniasis in Fuenlabrada. Madrid Actas Dermosifiliogr. 2013;104(4):334–42. 10.1016/j.adengl.2013.03.005. 23567452 10.1016/j.adengl.2013.03.005

[34] Gaspari V, Zaghi I, Macrì G, Patrizi A, Salfi N, Locatelli F, et al. Autochthonous cases of mucosal leishmaniasis in Northeastern Italy: clinical management and novel treatment approaches. Microorganisms. 2020;8(4):588. 10.3390/microorganisms8040588. 32325735 10.3390/microorganisms8040588 PMC7232153

