# Supplementary material for: Combination of Cpb-Hsp70 typing methods reveals genetic divergence between Leishmania infantum strains causing human tegumentary leishmaniasis in northern Italy and central Spain: a retrospective study
Source: Infect Dis Poverty. 2025 May 26;14:41. doi: 10.1186/s40249-025-01309-5 (PMC12105401; doi:10.1186/s40249-025-01309-5)
Supplement: Supplementary file 2 — Additional file 2. Figure S1. UV light visualization of electrophoretic run of Cpb-generated amplicons; Figure S2. UV light visualization of electrophoretic run of Hsp70 N-fragment (593 bp) generated amplicons. [file 40249_2025_1309_MOESM2_ESM.docx]

**Supplementary Figure 1.**

**
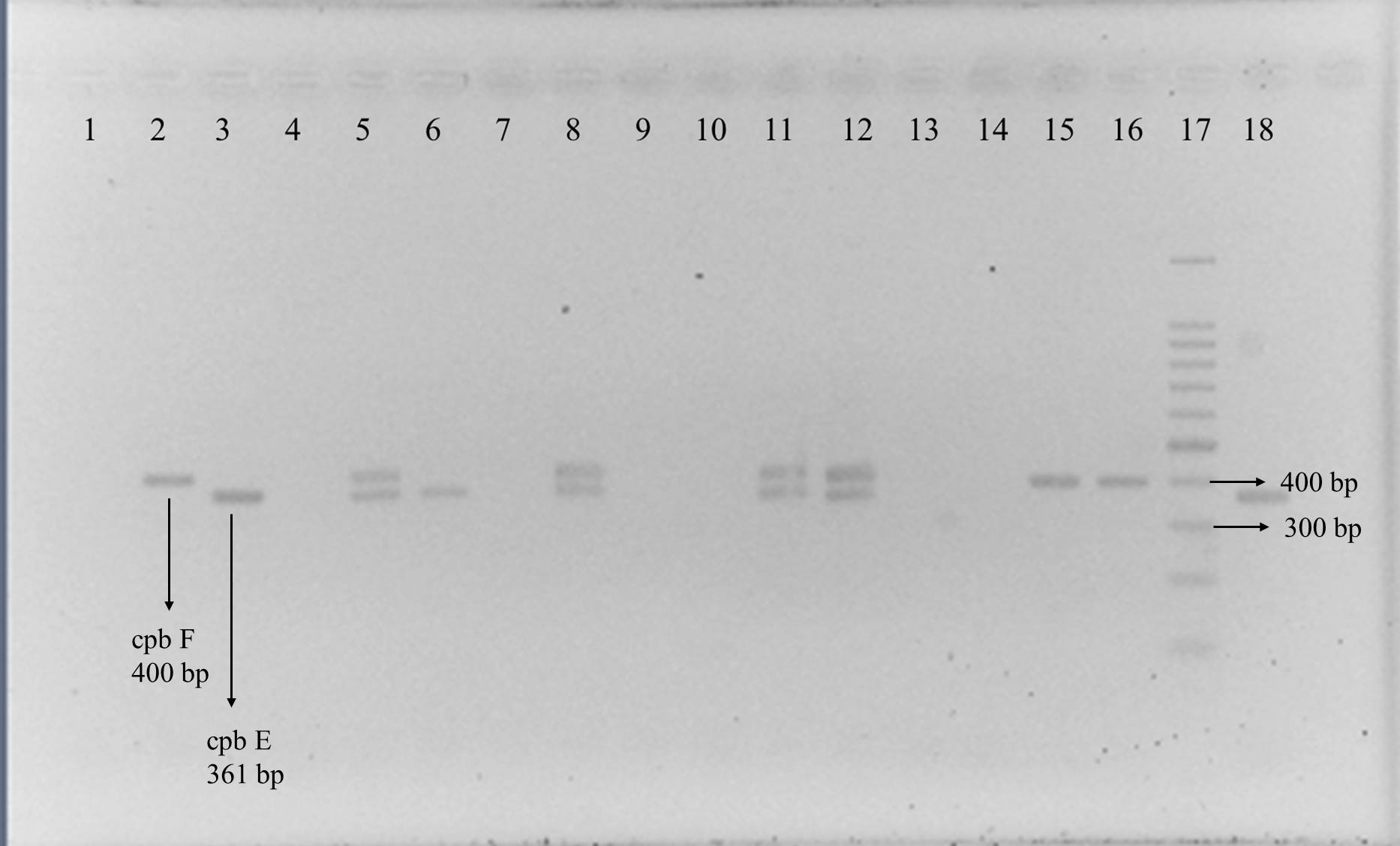
**

**UV light visualization of electrophoretic run of *Cpb*-generated amplicons.** Lane 1; negative control. Lane 17: DNA ladder (Promega, Madison, Wisconsin, United States) ranges in size from 100bp to 1,000bp in 100bp increments with an additional band at 1,500bp (on the top) . Lanes 2 and 16; positive controls for *Leishmania donovani*, ie *Cpb* amplicons obtained from gDNA of cultured promastigotes of the reference *L. donovani* strain LMHOM/IN/80/DD8 (displaying *Cpb* type F, 400 bp long). Lanes 3 and 18; positive controls for *L.infantum*, ie *Cpb* amplicons obtained from gDNA of cultured promastigotes of the reference *L. infantum* strain MHOM/TN/1980/IPT1 (exhibiting *Cpb* type E, 361 bp long). From lane 4 to lane 15 are shown amplicons, when present, obtained from 12 TL samples from Bologna (respectively: BO142, BO143, BO145, BO148, BO147, BO153, BO23 (a), BO23(b), BO156, BO154, BO155, BO158). Electrophoresis was run on 4% Agarose/TAE gel, staining was performed by soaking in a solution containing ethidium bromide (EtBr) (0.5µg/ml EtBr in water) after an electrophoretic run of 30 minutes.

**Supplementary Figure 2.**

**
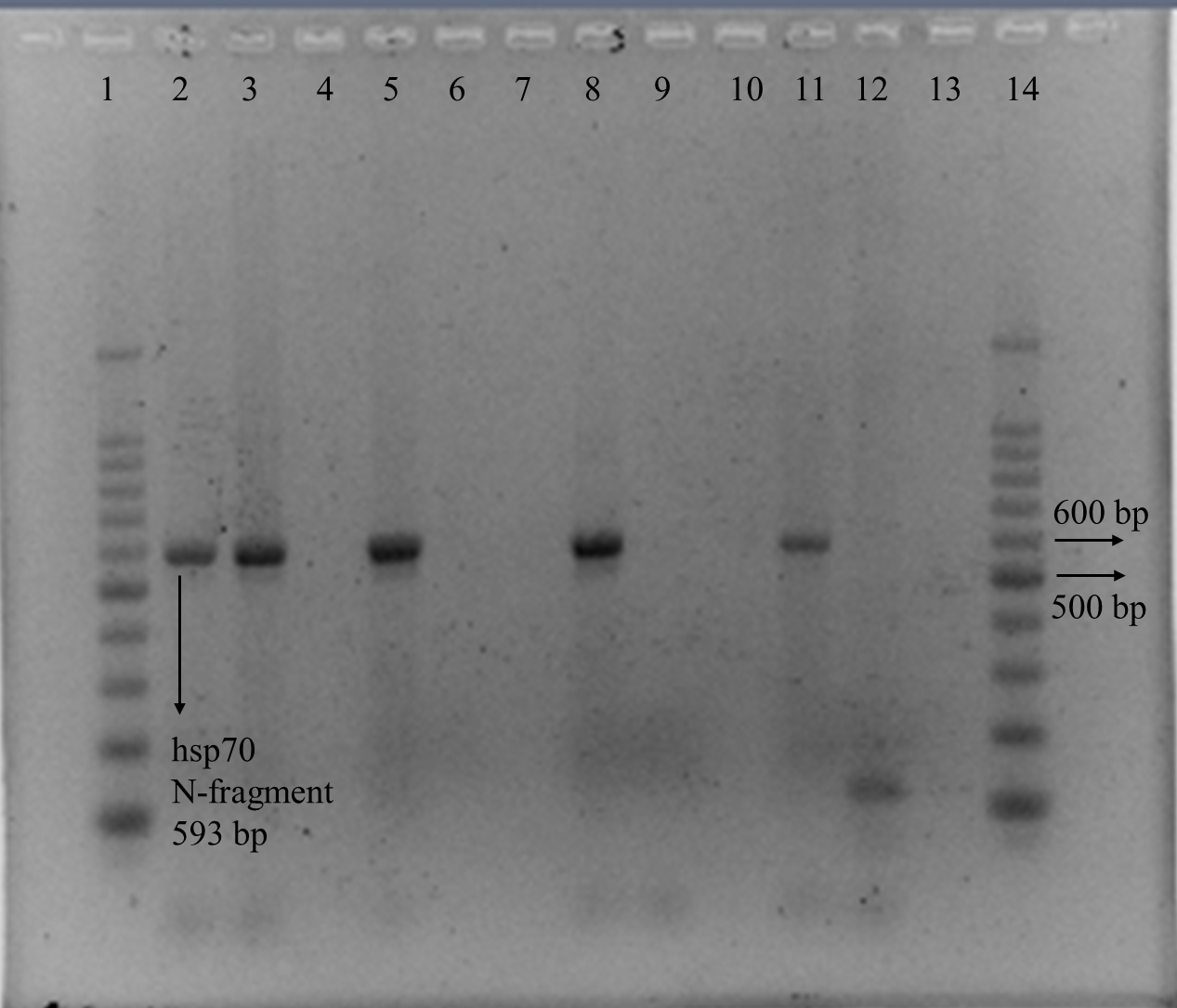
**

**UV light visualization of electrophoretic run of *Hsp70* N-fragment (593 bp) generated amplicons.** DNA ladder (Promega, Madison, Wisconsin, United States) ranges in size from 100bp to 1,000bp in 100bp increments with an additional band at 1,500bp (on the top) is shown on lane 1 and lane 14. Lane 2; positive control (amplification of N-fragment of hsp70 obtained from gDNA of cultured promastigotes of the reference *L. infantum* strain MHOM/TN/1980/IPT1). Lane 13; negative control. From lane 3 to lane 12; amplicons obtained from 10 Bologna samples (respectively, BO143, BO141, BO148, BO115(b), BO157, BO158, BO165, BO166, BO168, BO167). Electrophoresis was run on 2% Agarose/TAE gel stained by GelRED ® (Biotium inc. Fremont, USA).
